# Conjunctival Benign Fibrous Histiocytoma in a 57-year-old man

**Published:** 2019

**Authors:** Nicoleta Popescu, G. Alina Gheorghe, P. Calin Tataru, Mihai Constantin

**Affiliations:** * Clinique Notre-Dame de Grâce, Charleroi, Belgium; ** Emergency Eye Hospital, Bucharest, Romania; *** OphtalmoEsterel, Saint-Raphaël, France

**Keywords:** neoplasias of the conjunctiva and cornea, benign fibrous histiocytoma, ocular surface reconstruction, amniotic membrane

## Abstract

**Purpose.** To report a case of benign fibrous histiocytoma of the conjunctiva involving the cornea, an uncommon ocular surface tumor.

**Methods.** A 57-year-old patient came in our service complaining of a progressively enlarging conjunctival mass temporally to the limbus and invading the adjacent cornea of the left eye.

**Results.** The approach consisted in surgical excision followed by cryotherapy on the edges and on the base of the excision site and amniotic membrane patch reconstruction of the ocular surface defect. Pathologic examination and immunohistochemistry were performed in order to establish the diagnosis. No recurrences appeared in 8 months of follow up.

**Conclusions.** Fibrous histiocytoma might be easily misdiagnosed as it is exceedingly rare. Complete resection with careful inspection of edges is advised. Cryotherapy at the base and borders of the resection site is recommended as both benign and malignant tumors can show recurrence. Amniotic membrane should always be regarded as an efficient option in reconstruction of broad surface defects after tumor resection.

**Abbreviations:** FH = fibrous histiocytoma, CIN = corneal intraepithelial neoplasia, SSCA = squamous cell carcinoma, AM = amniotic membrane, MMC = topical mitomycin-C, 5-FU = 5-fluorouracil, BCVA = best corrected visual acuity

## Introduction

Fibrous histiocytoma (FH) is a mesenchymal tumor, considered amongst the most frequent soft tissue lesions. It usually appears as a slow growing nodule consisting of a variety of fibroblastic and histiocytic cells, blood vessels and collagen, which are often arranged in a cartwheel or storiform pattern [**1**]. The tumor can be either benign or malignant [**2**].

Ocular fibrous histiocytomas can involve different structures such as conjunctiva, corneoscleral limbus, cornea, eyelids or the orbit [**1**]. We chose to report this specific case because they are extremely rare tumors, only few cases being presented in literature [**2**-**9**]. 

Differential diagnosis is important and neoplastic lesions such as corneal intraepithelial neoplasia (CIN), squamous cell carcinoma (SSCA), amelanotic melanoma and conjunctival lymphoma should always be suspected. FH can also be misdiagnosed as pterygium, chalazia, leiomyoma, or nodular episcleritis [**10**].

Treatment consists in complete surgical resection with at least 3-4mm tumor-free margins followed by local cryotherapy at the excision base and borders and, if needed, appropriate ocular surface reconstruction [**10**]. Limbal lesions are managed by localized alcohol corneal epitheliectomy, main mass removal by a partial lamellar corneal scraping together with the involved conjunctiva, supplemental cryotherapy and reconstruction of the surface defect [**11**]. It is essential to let at least one quarter of normal limbal stem cells untouched in order to re-epithelize the cornea, otherwise a limbal stem cells transplant must be considered [**11**]. Amniotic membrane (AM) transplantation has become increasingly popular over the last years, with encouraging results in the reconstruction of conjunctival and corneal defects created by the surgical removal of tumors [**12**,**14**]. Topical mitomycin-C (MMC), 5-fluorouracil (5-FU) or interferon (IFNα2b) should be considered in recurrent, large or annular limbal lesions where substantial surgical excision may compromise the ocular surface [**11**]. Lifelong follow-up is essential as recurrence can occur, especially in malignant cases where both local and distant metastases can appear [**6**].

## Materials and Methods

**Fig. 1:**
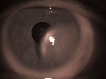
Conjunctival lesion invading the limbus and cornea passing over the pupillary margin

A 58-year-old male presented with a conjunctival mass in his left eye. The lesion was located temporally at the limbus, invading the adjacent cornea and measuring approximately 8mm (**Fig. 1**). 

**Fig. 2:**
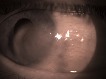
Slightly engorged conjunctival vessels passing over the conjunctival mass

The tumor was firm, slightly elevated and pinkish in color, with surrounding engorged conjunctival vessels. Superficial vessels were passing over the lesion (**Fig. 2**). 

Approximately 3.5 clock hours were invaded and deep central cornea was affected. This resulted in poor visual acuity with a best corrected visual acuity (BCVA) of 1/10. According to the patient, the lesion had been progressively extending over a period of one year.

No prior ophthalmic or medical histories were reported and, on systemic examination, no other nodules were noticed. The patient had been experiencing itching and foreign body sensation over the last 5 months for which he had been treated with lubricants. BCVA in the congener eye was 9/10 and biomicroscopic exam was within normal limits.

## Results

Resection of the mass was performed and the piece was sent for histopathologic examination, the tumor being as large as 4mm x 8mm. For the corneal part, absolute alcohol was applied for 30 seconds with subsequent balanced salt solution irrigation. This was followed by epithelial debridement and corneal scraping approximately 1.5mm into healthy cornea [**11**]. The limbal and conjunctival components were removed with 2,5mm safety margins, taking care of the muscle insertion. Adjunctive cryoapplications were applied at the borders of the resection site. As the surface defect was significantly large, we used an amniotic membrane (AM) patch for reconstruction. The AM was attached to the stromal side by interrupted 10.0 nylon sutures with the patch oriented towards the defect.

The pathologic examination and immunohistochemistry described a conjunctiva with the lamina propria containing a nodular mesenchymal proliferation of spindle cells and histiocytes without atypia, arranged in a storiform pattern, within a loose connecting stroma with slender collagen fibers, several inflammatory cells, and a fine capillary network. The overlying conjunctiva had limited erosions. The conclusive diagnosis was benign fibrous histiocytoma.

Eight months postoperatively, there was minimal conjunctival and corneal scarring at the surgical site, with no recurrence.

## Discussion

Even though fibrous histiocytoma is a common soft tissue tumor, its ocular incidence is exceedingly rare. The largest number of cases was reported in Kim et al. study, in which 6 cases of FH were analyzed. The results of the study showed a greater incidence in males than in females (5 men out of 6 patients) [**6**]. The average age was 37, medium tumor basal diameter was 7mm and there were 4 benign lesions and 2 malignant ones, with only one recurrence in a malignant case [**6**]. Two out of the total number of 35 that we found reported in literature were associated with Xeroderma Pigmentosum in children [**2**-**9**]. Varma P et al. made the first summary of all benign cases in 2014 and concluded that the mean age was 39 with no gender predilection [**3**]. Moreover, 74% were located at the corneoscleral limbus with a rate of recurrence of 20% for the BFH [**3**].

Suspicion of corneal and conjunctival intraepithelial neoplasia (CIN), squamous cell carcinoma (SSCA), amelanotic melanoma or conjunctival lymphoma should always be kept in mind, especially in elderly patients. Many of these lesions can only be diagnosed on pathologic examination, despite their history and clinical presentation [**11**]. In our case, we suspected more a CIN, which is why excessive care of resection with safety margins was made despite the site and size of the tumoral mass.

Complete resection of the tumor with safety margins is essential as local recurrence and metastasis typically develop after incomplete excision [**3**]. Additionally, the piece should be sent to histopathology for the diagnosis and to exclude a malignant invasion, which should be followed up more carefully. Cryotherapy is recommended to ensure a clear destruction of tumoral cells and prevent recurrence although no standard protocol has ever been proposed.

Reconstruction of the ocular surface should be considered whenever necessary. Amniotic membrane transplantation has proved to be successful in various ocular conditions such as persistent epithelial defects, corneal ulcers, and wide conjunctival defects after resection of lesions [**13**,**15**]. Moreover, its application goes beyond ophthalmology in different reparative and reconstructive procedures, as it contains abundant growth factors and anti-inflammatory proteins, promoting re-epithelialization, inhibiting fibrosis, and suppressing inflammation [**12**,**15**]. In our case, because the surface defect was significantly large, we considered amniotic membrane transplantation the best solution.

**Sources of funding**

The authors received no financial support for the research, authorship, and/ or publication of this article.

**Acknowledgements**

All the authors have equally contributed to this paper.

**Disclosures**

The authors have no conflict of interest to declare.
